# Clean thermal decomposition of tertiary-alkyl metal thiolates to metal sulfides: environmentally-benign, non-polar inks for solution-processed chalcopyrite solar cells

**DOI:** 10.1038/srep36608

**Published:** 2016-11-09

**Authors:** Jungwoo Heo, Gi-Hwan Kim, Jaeki Jeong, Yung Jin Yoon, Jung Hwa Seo, Bright Walker, Jin Young Kim

**Affiliations:** 1 Department of Physics and Applied Mathematics, Ulsan National Institute of Science and Technology (UNIST), Ulsan 689-798, South Korea; 2 Department of Energy Engineering, School of Energy and Chemical Engineering, Ulsan National Institute of Science and Technology (UNIST), Ulsan 689-798, South Korea; 3 Department of Materials Physics, Dong-A University, Busan, 604-714, South Korea

## Abstract

We report the preparation of Cu_2_S, In_2_S_3_, CuInS_2_ and Cu(In,Ga)S_2_ semiconducting films via the spin coating and annealing of soluble tertiary-alkyl thiolate complexes. The thiolate compounds are readily prepared via the reaction of metal bases and tertiary-alkyl thiols. The thiolate complexes are soluble in common organic solvents and can be solution processed by spin coating to yield thin films. Upon thermal annealing in the range of 200–400 °C, the tertiary-alkyl thiolates decompose cleanly to yield volatile dialkyl sulfides and metal sulfide films which are free of organic residue. Analysis of the reaction byproducts strongly suggests that the decomposition proceeds via an SN_1_ mechanism. The composition of the films can be controlled by adjusting the amount of each metal thiolate used in the precursor solution yielding bandgaps in the range of 1.2 to 3.3 eV. The films form functioning p-n junctions when deposited in contact with CdS films prepared by the same method. Functioning solar cells are observed when such p-n junctions are prepared on transparent conducting substrates and finished by depositing electrodes with appropriate work functions. This method enables the fabrication of metal chalcogenide films on a large scale via a simple and chemically clear process.

Since their discovery, solar cells have been recognized as an appealing energy source due to the abundance of solar energy, their simplicity and lack of moving parts and their ability to generate power in remote locations. Although the practical use of solar modules in the past has been confined to niche applications (where their high cost could be justified), the aggregate amount of energy produced by solar modules has increased by more than one thousand times over the past 20 years and power generation from photovoltaics now constitutes approximately 1% of global electrical energy production^1^. As other energy sources become increasingly scarce and the burning of fossil fuels is curbed to mitigate climate change^2^, the impetus to produce energy from sunlight continues to grow stronger every year. The large scale production of solar energy requires the deployment of solar cells over large surface areas, which is economically problematic due to the relatively high cost per area to manufacture photovoltaic modules. Thus, finding low-cost materials and strategies to fabricate solar cells constitutes an immensely important scientific challenge facing our generation.

Recent years have witnessed the development many new types of solar technologies which can be deposited like inks by low cost solution coating techniques including organic bulk heterojunction (BHJ) solar cells^3^, dye-sensitized solar cells (DSSC)^4^, colloidal quantum dot (CQD) solar cells^5^ and perovskite solar cells^6^. Although the power conversion efficiencies (PCEs) of these technologies have developed rapidly to approach those of their inorganic counterparts, their stabilities and reliabilities remain unresolved.

Another route to accomplish the same goals as BHJ, DSSC, CQD and perovskite approaches is to develop strategies to prepare more traditional inorganic solar cells by solution processing methods. CuInS_2_^7^ and related Cu(In,Ga)Se_2_ materials have been shown to yield PCEs of over 20%^8^,^9^, as high as any solar cell based on BHJ, DSSC, CQD or perovskite materials, while the long-term stability of these modules has already been established. Although these chalcogenide materials are generally insoluble, it has been demonstrated that using appropriate chemistry, soluble precursor compounds can be prepared and processed like inks^10^,^11^. For instance, Mitzi and co-workers have shown that Cu(In,Ga)Se_2_ cells can be fabricated by processing films from solutions of hydrazinium metal selenides (using hydrazine as a solvent) followed by thermal annealing and selenization of the precursor films^12^. Although this has proven to be an effective method to prepare Cu(In,Ga)Se_2_ type solar cells, hydrazine is a toxic, corrosive and chemically reactive solvent. Alternatively, Cu(In,Ga)Se_2_ nanoparticles (NPs) can be synthesized and deposited by solution processing^13^,^14^,^15^. However, these NP based devices typically lead to low PCEs without a subsequent high temperature selenization step.

The ultimate goal of researching solution-processed solar cells is to enable the manufacture solar cells on a large scale using industrial printing equipment. In the context of large-scale printing operations, inks based on non-toxic and non-polar solvents offer the best performance due to their low reactivity, low surface tension, good wettability, evaporation rates and ability to form smooth and uniform films. Thus, the ability to process solar cells using non-toxic, non-polar solvents offers the best compatibility with industrial-scale printing equipment.

There are few examples of CuInS_2_ or Cu(In,Ga)Se_2_ solar cells being deposited from precursors using common organic solvents^16^,^17^,^18^,^19^,^20^. Until now, these works have utilized metal xanthate precursors which must be prepared using carbon disulfide, which poses health and reactivity problems like hydrazine. Metal xanthates additionally possess undesirable reactivity; they are very sensitive to pH and yield an ill-defined a mixture of reactive products when they decompose. Other works^21^,^22^ have utilized mixtures of metal halides and thiourea instead of single molecule precursors, however, it is unclear what happens to the halide anions or thiourea decomposition products after the metal sulfides are formed. Thus, molecular precursors which decompose by a well-defined reaction to yield well-defined products have until now not been demonstrated.

In this contribution, we have used a rational, organo-chemical approach to design metal complexes which are soluble in non-toxic, non-polar solvents and decompose thermally to yield pure metal sulfide films. The t-butoxy carbonyl (TBOC) group was used as a model and starting point in the design of these complexes; the TBOC moiety is well-known to decompose quantitatively via SN_1_ or E_1_ mechanisms at elevated temperatures to yield volatile iso-butene and CO_2_ byproducts^23^. The thermally-labile TBOC moiety has been successfully exploited as a solubilizing group to solution-process organic semiconductor films which become crystalline and insoluble after heating, allowing high-mobility, multilayer films to be processed^24^,^25^,^26^. In this work, we have extended this concept to solution-process insoluble, inorganic metal sulfide films. We show that tertiary-alkyl metal thiolates (TAMTs) decompose analogously to the TBOC group to yield volatile alkyl sulfides and insoluble metal sulfide films which are free from organic residue.

We show that CuInS_2_ and Cu(In,Ga)S_2_ films can be readily prepared by annealing TAMT films which are deposited from solutions in common organic solvents, as illustrated in Fig. 1. Films with a range of compositions can be prepared by adjusting the ratio of each TAMT (Cu, In, and Ga) in order to achieve a range of band gaps and allowing detailed control of the energy band structure. These films can be deposited on conductive transparent indium tin oxide (ITO) substrates, with appropriate electrodes, in order to produce photovoltaic devices (Fig. 2). By grading the bandgap of the active layer, it is possible to create a band structure which drives photoexcited electrons from the absorbing layer to the p-n junction.

This report marks the first of its kind to utilize rationally designed CuInS_2_ precursors which thermally decompose via a well-understood reaction. The complexes yield volatile by-products with low reactivity (dialkyl sulfides) and metal sulfide films which are free of organic residue. The simple reagents used, coupled with the straightforward synthesis and processing of the materials make this approach well-suited for large scale solar cell production.

## Results

### Synthesis

**Figure 3:**
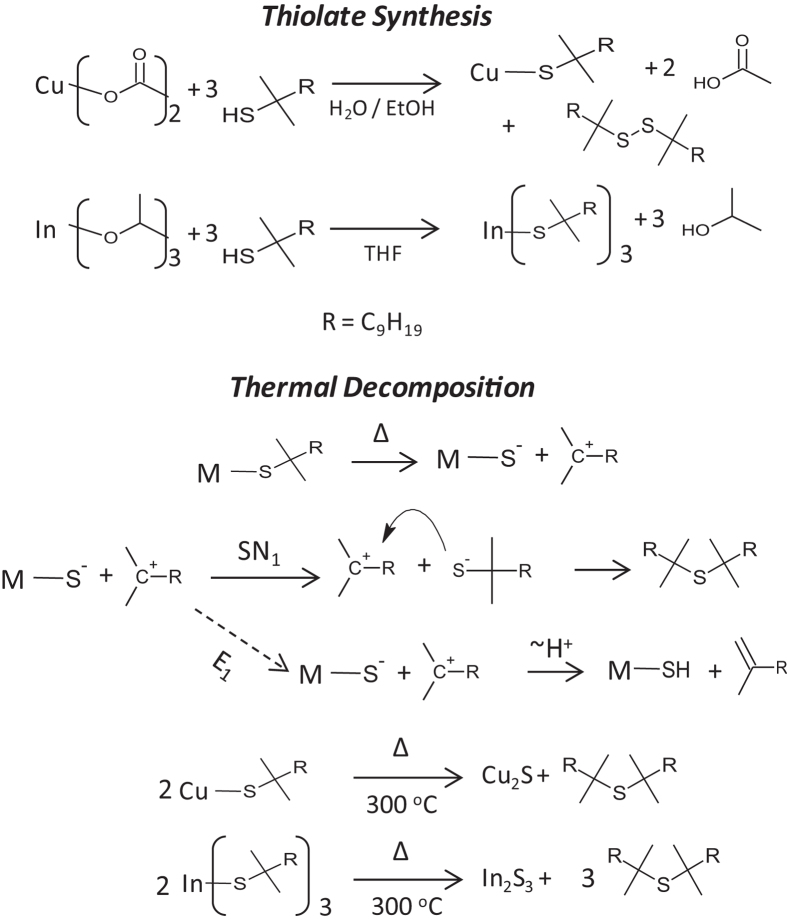
Synthesis of metal thiolates and their thermal decomposition to metal sulfides.

The TAMT materials were conveniently synthesized by reacting metal alkoxides or acetates with tertiary alkane thiols as shown in Fig. 3. In the case of copper t-dodecanethiolate (CuTDT), Cu(II) acetate reacted with 2 equivalents of t-dodecanethiol, undergoing a reduction/oxidation reaction with simultaneous anion displacement to afford the Cu(I) thiolate, with acetic acid and t-dodecyl disulfide as byproducts. Ga and In isopropoxides were found to readily react with t-dodecanethiol; isopropoxide is a strong base which is quickly neutralized by proton transfer from t-dodecanethiol (a weak acid) to yield Ga and In t-dodecanethiolates (GaTDT and InTDT) and isopropanol. The isolated TAMTs were found to be insoluble in polar solvents including water and methanol, but exhibited excellent solubility in non-polar solvents and solvents with intermediate polarity including hexane, toluene, chloroform and tetrahydrofuran (THF). The TAMTs were found to be stable for months at room temperature in the absence of light, but decomposed rapidly to metal sulfides upon heating to moderate temperatures.

### Thiolate Decomposition

**Figure 4:**
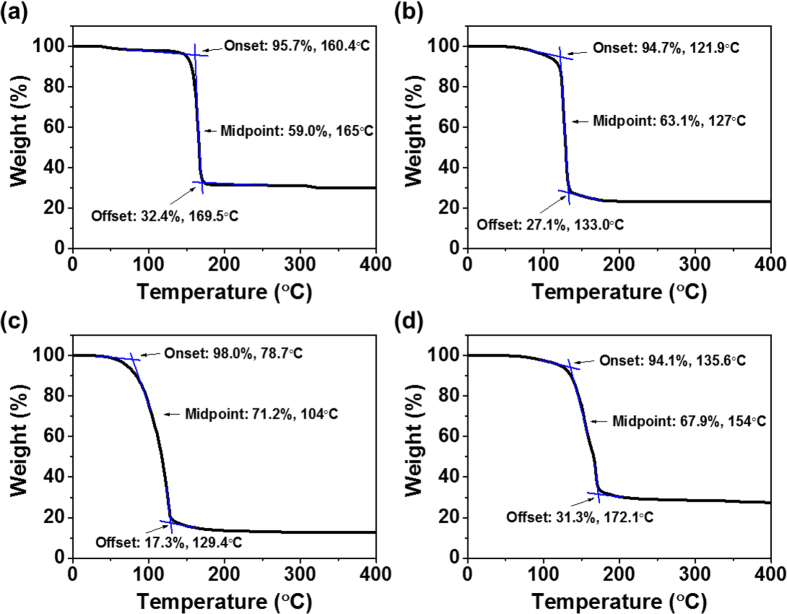
TGA plots of (**a**) CuTDT, (**b**) InTDT, (**c**) GaTDT, and (**d**) a CuTDT and InTDT 1:1 molar mixture, respectively.

In order to quantify the thermal decomposition of the precursors into metal sulfides, thermogravimetric analysis (TGA) was performed. Figure 4 shows TGA traces of each thiolate complex as well as a 1:1 molar mixture of CuTDT and InTDT. CuTDT begins to decompose at 160.4 °C (95.7% of original weight) and rapidly loses mass to 32.4% of its original weight during an increase of 9.1 °C. InTDT decomposes to 27.1% of its original weight in the range of 121.9 °C to 133 °C, while GaTDT steadily decomposes to 17.3% of its original weight starting at 78.7 °C and ending at 129.4 °C (a range of 50.7 °C).

The 1:1 molar mixture of CuTDT and InTDT converts to the metal sulfide phase somewhat more gradually over a range of 46.5 °C, losing 62.8% of its weight without showing two distinct decomposition steps. The losses in mass are consistent with the thermal decomposition mechanisms outlined in Fig. 3. Although all materials decompose to metal sulfides below 200 °C, TAMT films were processed at a reference temperature of 300 °C throughout this study to ensure complete conversion of the insulating precursors to semiconducting sulfide films. For some experiments, higher annealing temperatures were used as noted in order to achieve better film crystallinity.

In order to confirm the decomposition mechanism and identity of the reaction by-products, we decomposed samples of the complexes by heating to 300 °C under vacuum and trapped the volatile decomposition products with a liquid nitrogen trap. The decomposition products were dissolved in chloroform and analyzed by gas chromatography -mass spectrometry (MS, see Supplementary Figure S1) and Raman spectrometry (Supplementary Figure S2).

A control sample consisting of the thiol starting material was analyzed as a reference, however, no peak corresponding to the MW of the parent thiol (M = 202) was observed in the MS spectrum and the largest MW observed in the thiol spectrum (M = 168) corresponded to the MW of the thiol – H_2_S, i.e. the alkene, indicating that the C-S bond of the thiol was completely fragmented during ionization in the MS instrument. This is consistent with reference MS spectra of t-dodecanthiol (2-methyl-2-undecanethiol) which show no peak at M = 202 corresponding the un-fragmented compound^27^. The decomposition products obtained by heating a mixture of CuTDT and InTDT also showed a highest MW of 168 with spectra which closely resembled the parent thiol. The MS results thus did not distinguish between alkylthiol, alkene or dialkyl sulfide, due to the ease with which the C-S bond is cleaved in the MS instrument, however, were consistent with the presence of any one of these compounds.

Raman spectra of the decomposition products (Figure S2) clearly showed that no alkene or thiol were present, but showed strong peaks corresponding to C-S vibrations, indicating that the volatile decomposition product consists chiefly of the dialkyl sulfide. This strongly suggests that the decomposition of the TAMT occurs primarily via an SN_1_ mechanism as shown in Fig. 3. Although we initially anticipated that the thiolates decomposed via an E_1_ mechanism, (dashed arrow, Fig. 3) the SN_1_ route is not unexpected given the presence of the thiolate anions in the films, which are known to be exceptionally strong nucleophiles and apparently react with the carbocation intermediate before proton transfer and elimination of the alkene can occur. The rapid, thermally activated decomposition at lower temperatures than primary or secondary thiolates^28^ is also consistent with E_1_ or SN_1_ processes and further substantiate the decomposition mechanism.

Raman spectra of CuInS_2_ films prepared by spin coating an equimolar ratio of CuTDT and InTDT and annealing at 200 °C and 300 °C are also shown in Figure S2. The spectra of films annealed at 200 or 300 °C show no bands in the range of 600–1200 cm^−1^ corresponding to C-S, C-S-C or CH_2_ vibrational modes and no sharp peaks in the range of 2400–3000 cm^−1^ corresponding to S-H or C-H vibrations, (Table S1) indicating that no detectable amounts of organic residue were present after annealing at either 200 or 300 °C. A plot of the un-annealed precursor is included for reference showing strong C-H stretching signals at ~2800 to 3000 cm^−1^ and several other sharp peaks in the range of 400 to 2500 cm^−1^ which disappear after annealing films at 200 °C or more. These results are consistent with the TGA measurements; upon heating the TAMTs, the mass decreases in the range of 120–170 °C and reaches a constant value, indicating that the organic constituents have completely volatilized and no organic residue is left at 200 °C.

The presence of CuInS_2_ was confirmed by examining the region of the Raman spectrum near 300 cm^−1^ (Figure S2). Phase-pure CuInS_2_ with the tetragonal chalcopyrite structure exhibits a Raman spectrum dominated by a sharp A1 signal at 290–292 cm^−1^ 29,30. A very broad peak at 298 cm^−1^ with a strong shoulder was observed for films annealed at 300 °C, consistent with the presence of disordered CuInS_2_. Upon annealing at a higher temperature of 570 °C (just below the softening temperature of the glass substrate), the Raman spectrum becomes much sharper while the relative intensity of the shoulder decreases, indicating that the tetragonal chalcopyrite phase crystallizes and that the crystal quality improves upon annealing at higher temperature. However, it is apparent that the crystallinity of films post-annealed in this way is still inferior to films which are epitaxially grown or sulfurized at high temperatures.

### Sulfide Film Characteristics

**Figure 5:**
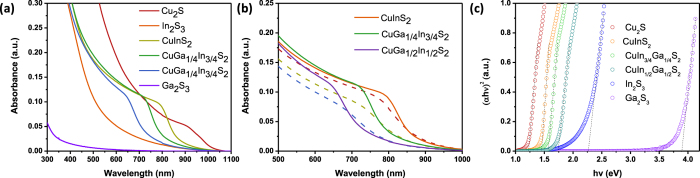
UV-vis spectra of sulfide films prepared on glass substrates. (**a**) Spectra of all materials annealed at 400 °C. (**b**) Zoomed plot of the 500–1000 nm spectral region showing absorption onsets for Cu(In,Ga)S_2_ materials annealed at 300 (dashed traces) or 400 °C (solid lines) (**c**) Tauc plots showing (αhν)^2^ vs hv (for direct optical transitions).

UV-vis absorption spectra of Cu_2_S, In_2_S_3_, and different compositions of Cu(In,Ga)S_2_ are shown in Fig. 5, while the optical properties are summarized in Table 1. Cu_2_S absorbs light up to 1020 nm optical energy gap (E_g_ (opt) = 1.22 eV), whereas In_2_S_3_ exhibits an absorption onset at 550 nm. (E_g_ (opt) = 2.25 eV). CuInS_2_ films were prepared by spin coating a mixed TAMT solution with 1:1 molar ratio, the CuInS_2_ absorption spectra exhibit a clear absorption onset which is distinct from either Cu_2_S or In_2_S_3_. This implies that a distinct CuInS_2_ phase is formed, as opposed to a mixture of Cu_2_S and In_2_S_3_ phases.

The presence of a distinct CuInS_2_ phase after annealing at 400 °C and 570 °C was confirmed by X-ray diffraction (XRD) (Supplementary Figure S3). Diffraction patterns of films annealed at both temperatures fit the tetragonal chalcopyrite phase, however, the diffraction peaks become much narrower at higher temperature. The full width at half maximum of the 112 plane decreased from 3.22 to 1.26 ° upon annealing at 400 and 570 °C, respectively, indicating that relatively amorphous CuInS_2_ material initially formed by the decomposition of the thiolates is able to crystallize in the tetragonal chalcopyrite phase when heated sufficiently, consistent with the trends observed in Raman spectra. Films annealed at 200 °C or 300 °C did not exhibit any significant diffraction pattern, indicating an amorphous structure and low crystallinity in films annealed at lower temperatures.

As expected, were able to tune the optical bandgap of CuInS_2_ by adding GaTDT to mixed CuTDT/InTDT solutions. This process leads to a widening of E_g_ in the Cu(In,Ga)S_2_ films, as Ga atoms replace In atoms in the structure. A 3:1 molar ratio of In:Ga in Cu(In,Ga)S_2_ film exhibits a blue-shifted absorption onset by 60 nm, while a 140 nm blue-shift is observed in the film with 1:1 molar ratio of In:Ga. In contrast, pristine Ga_2_S_3_ films show virtually no absorption of visible light due to the wide bandgap of Ga_2_S_3_ (3.26 eV). Figure 5b shows the effect of annealing temperature on the absorption spectra of Cu(In,Ga)S_2_ films. As we confirmed in TGA, TAMTs could be converted to metal sulfides below 200 °C. However, films were annealed at 300 or 400 °C to encourage crystallization and to obtain suitable absorption data; it is clear that films annealed at 400 °C show sharper absorption onsets and higher optical density than films annealed at 300 °C. For comparison, absorption data were plotted as Tauc plots of (αhν)^½^ vs hν (Supplementary Figure S4) and (αhν)^2^ vs hν (Fig. 5c) to model indirect and direct optical transitions, respectively. The plots of (αhν)^½^ vs hν indicate similar E_g_ values as obtained by fitting absorption onsets. The (αhν)^2^ plots showed good fits as well, except for In_2_S_3_, which has been shown to have both direct and indirect optical transitions, causing an indistinct absorption onset which is consistent with previous observations^31^,^32^.

### Film Morphology

**Figure 6:**
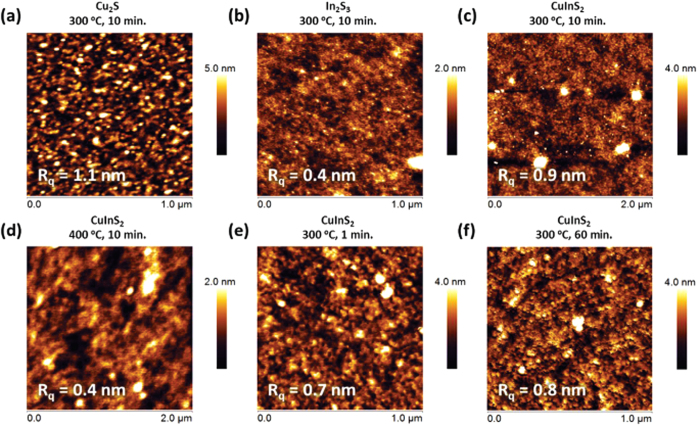
AFM images showing (**a**) Cu_2_S, (**b**) In_2_S_3_ and (**c**) CuInS_2_ films prepared by annealing the corresponding metal thiolates at 300 °C for 10 minutes. Image (**d**) shows a CuInS_2_ film annealed at a higher temperature of 400 °C while images (**e**) and (**f**) show films annealed at 300 °C for 1 and 60 minutes, respectively.

The surface morphology of each film was characterized by atomic force microscopy (AFM). AFM images of Cu_2_S, In_2_S_3_, and CuInS_2_ are shown in Fig. 6. All films show smooth surfaces with RMS roughness values of 1.1 nm or less. Figure 6(a–c) shows the surface morphology of Cu_2_S, In_2_S_3_, and CuInS_2_, respectively, processed at 300 °C for 10 min. Cu_2_S exhibits a rougher and irregular morphology compared to In_2_S_3_ and CuInS_2_ films, with RMS roughnesses of 1.1, 0.4 and 0.9 nm, respectively. CuInS_2_ shows intermediate surface features compared to Cu_2_S and In_2_S_3_. In the case of CuInS_2_ annealed at 400 °C, (Fig. 6d), the horizontal size of the surface features becomes larger than films annealed at 300 °C, however, the surface morphology becomes smoother. Figures 6c,e,f compare how varying the annealing time from 1 to 60 minutes affects morphology. These images suggest that annealing time does not greatly affect to the surface morphology of the films. To confirm the surface morphology of the CuInS_2_ films, scanning electron micrograph (SEM) images were taken of the surface. An SEM image of a CuInS_2_ film annealed at 300 °C for 10 minutes is shown in Supplementary Figure S5, revealing a smooth and amorphous surface, consistent with the AFM images.

**Figure 7:**
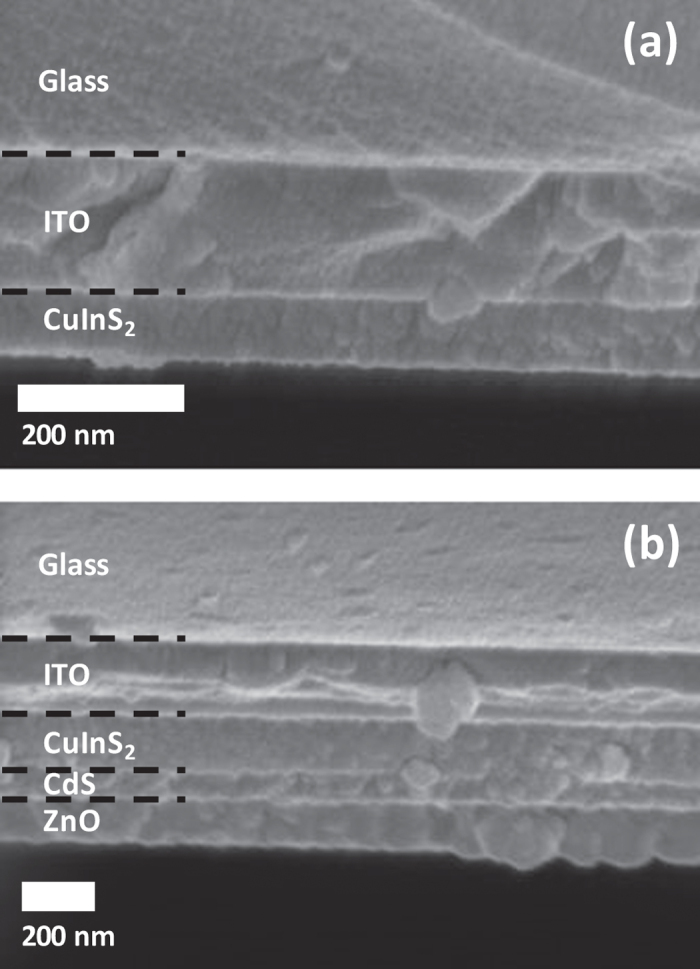
SEM images showing (**a**) a CuInS_2_ film deposited on a glass/ITO substrate and (**b**) a device stack consisting of a CuInS_2_/CdS/ZnO trilayer structure deposited on a glass/ITO substrate. CuInS_2_ and CdS layers were annealed at 300 °C, while ZnO was annealed at 120 °C.

SEM images were collected for cross-sections of CuInS_2_ films and CuInS_2_/CdS/ZnO trilayer films prepared on glass/ITO substrates were collected as well. These images are shown in Fig. 7. It can be seen that films are smooth and continuous, and that the CuInS_2_ films are approximately 80 to 100 nm thick.

### Electronic Properties

**Figure 8:**
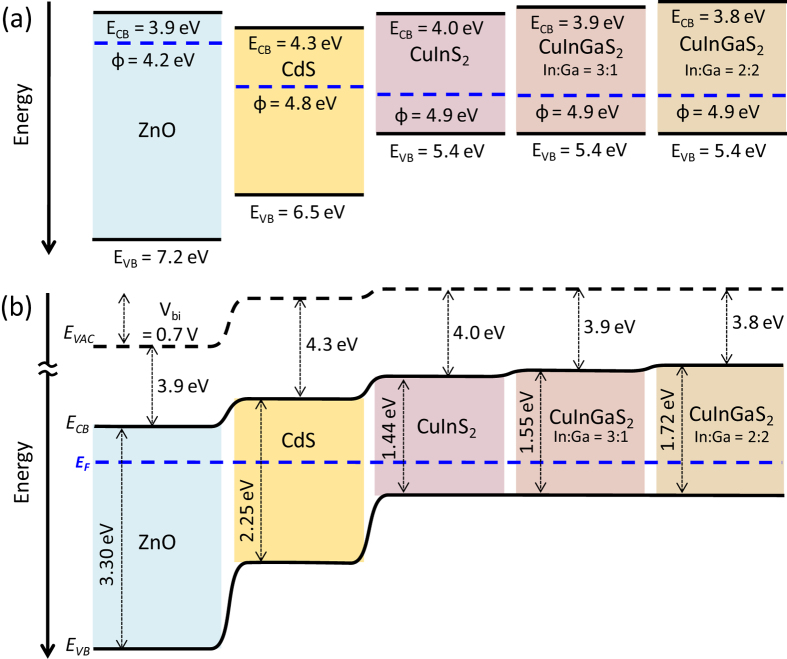
Energy band diagrams. (**a**) Absolute work function, valence and conduction band energies of each material. (**b**) Band diagram constructed from UPS and UV-vis absorption data.

X-ray photoelectron spectra (XPS) were collected for Cu_2_S, In_2_S_3_ and CuInS_2_ films (Supplementary Figure S6) confirming the presence of Cu in Cu_2_S and CuInS_2_ films and confirming the presence of In in In_2_S_3_ and CuInS_2_ films. Sulfur was present in all the films and the binding energies of the peaks were consistent with metal sulfide bonding states in all samples. Ultraviolet photoelectron spectra (UPS) data (Supplementary Figure S7) and UV-vis absorption spectra (Fig. 5) were used to construct energy band diagrams as shown in Fig. 8. Work functions (φ) were calculated from the secondary edge onsets (ESE) of each spectrum via the equation φ = 21.2 eV - ESE. The work functions were found to be 4.9 eV for all of the samples with the composition Cu(In,Ga)S_2_, compared to 4.8 and 4.2 eV previously reported for CdS and ZnO^33^. Varying the content of Ga element in CuInS_2_ film did not affect to the work functions of the materials. Valence band edge energies (E_VB_) of each layer were obtained from the Fermi edge relative to an Au reference. We completed the energy band diagram deducing the level of the conduction band edge (E_CB_) of each film from the collected UV-vis absorption data. ZnO and CdS layers showed deep E_VB_s of 7.2 eV and 6.5 eV, respectively, while three different Cu(In,Ga)S_2_ films exhibit the same E_VB_ at 5.4 eV. This implies that the different absorption onsets of these films can be ascribed to changes in E_CB_ due to Ga incorporation.

### Solar cell devices

**Figure 9:**
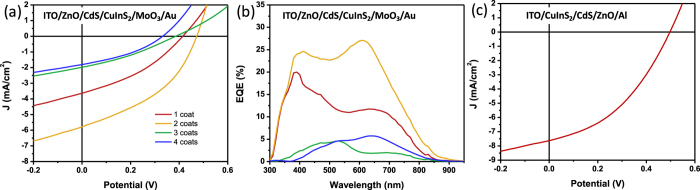
Solar cell characteristics. (**a**) J-V characteristics and (**b**) EQE spectra of CuInS_2_ solar cells prepared on ZnO/CdS substrates. (**c**) J-V characteristics of solar cells prepared on ITO substrates.

Solar cells were prepared by spin coating and annealing TAMTs onto conductive, transparent ITO substrates with an ITO/ZnO/CdS/CuInS_2_/MoO_3_/Au structure. CuInS_2_ layers were spin cast with 1:1 molar ratio of CuTDT and InTDT and annealed at 300 °C for 45 min. CdS window layers were also fabricated from Cd thiolate precursor following a similar method developed by our group^20^. Current density versus potential (J-V) characteristics of different thickness of CuInS_2_ solar cells are shown in Fig. 9a. We controlled the thickness of the CuInS_2_ layer by varying the number of coats. Using an overall concentration of 100 mg/mL in THF and a spin rate of 1500 rpm, a thickness of ~80–100 nm is obtained for each coat. Devices with 2 coats of CuInS_2_ layer showed better current density (J_SC_) and open circuit voltage (V_OC_) compared to devices with 1 coat of CuInS_2_. However, further increases in the thickness of the CuInS_2_ layer caused a decrease in performance. We presume that the annealing temperature of 300 °C was not sufficient to fully crystallize the CuInS_2_ phase, which leads to poor carrier extraction in thick films. A decrease in V_OC_ in the device with more than 2 coats of CuInS_2_ indirectly verifies an increase in the charge carrier recombination rate in thicker CuInS_2_ layers.

The solar cell characteristics of the device using an active layer comprising two coats of CuInS_2_ include a J_SC_, V_OC_, FF and PCE of 5.8 mA/cm^2^, 0.472 V, 0.408 and 1.11%, respectively. Figure 9b shows the external quantum efficiency (EQE) of the devices. We expected that the enhanced absorption of the thicker CuInS_2_ layer would increase the J_SC_. However, the EQE was significantly reduced after 2 coats of CuInS_2_. This indicates that the reduced EQE can be attributed to lowered internal quantum efficiency of the device, or poor charge carrier extraction due to the ill-defined crystalline structure confirmed by Raman (Figure S2) and XRD patterns (Figure S3). The different spectral shape of the EQE depending on the thickness of the devices arises from optical interference effects inside the active layer due to the thickness of the active layer, which is comparable to the wavelengths of incident light. Although such optical interference effects are unusual in CIGS devices (which are typically thicker than 1000 nm) they are common in devices with thin active layers such as organic and perovskite solar cells. Devices were prepared in an inverted architecture by depositing the CuInS_2_ film (two coats) first onto glass/ITO substrates, followed by deposition of CdS and ZnO layers. A J-V curve corresponding to this device architecture is shown in Fig. 9c. The performance in this architecture was somewhat improved, including a J_SC_, V_OC_, FF and PCE of 7.4 mA/cm^2^, 0.497 V, 0.403 and 1.53%. These PCE values are comparable to other CuInS_2_ devices prepared by solution processing methods such as NPs or other precursors without a high-temperature annealing or selenization step^13^,^14^,^34^, however, are low compared to CuInS_2_ or Cu(In,Ga)Se_2_ devices which are grown epitaxially or selenized at high temperature. Although the films are chemically pure, the relatively low PCE and thin optimum active layer thickness are consistent with a disordered film structure with small crystal domains, where abundant grain boundaries lead to short minority carrier diffusion lengths and limit the active layer thickness. Initial attempts to increase the annealing temperature to 400 °C unfortunately led to a dramatic drop in V_OC_ and high rate of device failure, which we presume may be due to instability between the ITO substrates and deposited chalcogenide layers at high temperatures. Although the band structure of the Ga-doped films indicates a slope in the conduction band which should provide an increased driving force pushing photogenerated electrons towards the p-n junction, initial attempts to incorporate Ga into the device structure yielded low J_SC_ values. We are currently investigating methods to improve the crystallinity of the materials using higher annealing temperatures with alternative substrates such as molybdenum, as well as post-deposition selenization, which we anticipate will allow much higher efficiencies.

## Discussion

Various metal thiolates (Cu, In, Ga) were synthesized as effective metal sulfide precursors through a low-cost and straightforward synthetic strategy. Spin coated films could be easily processed using non-polar and non-toxic solvents and decomposed cleanly at moderate temperatures to yield films free of organic residue. The volatile decomposition products were identified as dialkyl sulfides indicating that the complexes decomposed via a well-defined SN_1_ mechanism, analogously to the TBOC group. Binary, ternary, and even quaternary systems of metal sulfide films were solution-processed using mixed TAMT precursor solutions by spin casting followed by mild thermal annealing. The versatility of this method was demonstrated using four different metal complexes. The films were characterized electronically and exhibited band structures consistent with semiconducting Cu_2_S, In_2_S_3_, CuInS_2_ and Cu(In,Ga)S_2_ films. Functional solar cells were fabricated using the same techniques and equipment used for polymer solar cells by successive deposition of metal sulfide layers. This report marks the first of its kind to demonstrate the preparation of environmentally friendly CuInS_2_ type solar cells from non-polar thiolate precursors which are soluble in common organic solvents. In contrast with the widely used epitaxial growth methods used to prepare metal chalcogenide films, this approach constitutes a rapid, low-cost, strategy to prepare metal chalcogenide thin films, which is compatible with large-scale deposition methods such as printing or roll-to-roll coating. It offers great potential for application in the fabrication of a range of large-area semiconducting devices such as solar cells, LEDs, photodiodes and transistors, as well presenting the possibility for use in conjunction with solution-processed organic and hybrid semiconducting devices.

## Methods

### Synthesis

All reagents were procured from Aldrich or Alfa Aesar and used as received. Unless otherwise noted, all materials were handled in a nitrogen filled glovebox or using standard Schlenk techniques. TAMT materials were stored in a nitrogen filled glovebox and protected from light by wrapping in aluminum foil. It should be noted that the thiols used in this work possess a very strong odor and should be handled carefully.

#### Copper t-dodecanethiolate (CuTDT)

Copper (II) acetate monohydrate (1.00 g, 5.00 mmol) was dissolved in a mixture of 12 mL ethanol and 12 mL water yielding a deep blue solution. A small amount of solid did not dissolve. t-dodecanethiol (2.78 mL, 11.8 mmol) was separately dissolved in 8 mL of ethanol. The two solutions were combined in a large vial and shaken for 15 minutes, during which time the liquid phase changed from blue to green to hazy yellow, indicating a reduction of Cu (II) to Cu (I), while the remaining copper acetate dissolved. The mixture was centrifuged at 2000 rpm for 5 minutes and a thick oily phase separated to the bottom. The supernatant liquid was discarded and the oily phase was washed several times with anhydrous methanol. The viscous material was transferred to a Schlenk flask and dried under vacuum overnight.

#### Indium t-dodecanethiolate (InTDT)

In a nitrogen-filled glovebox, indium isopropoxide (0.584 g, 2.0 mmol) was weighed into a 10 mL vial and dissolved in 4 mL of anhydrous THF. T-dodecanethiol (1.55 mL, 6.6 mmol) was added to the solution and shaken vigorously. The mixture was allowed to stand at room temperature for 2 hr. before being filtered through a 0.45 micron PTFE syringe filter into a Schlenk flask. The solvent and excess thiol were removed under vacuum and the resulting viscous clear material was dried under vacuum overnight.

#### Gallium t-dodecanethiolate (GaTDT)

The same procedure as used for InTDT was used, with the exception that gallium isopropoxide (0.494 g, 2.0 mmol) was used instead in place of indium isopropoxide.

#### Cadmium t-nonanethiolate (CdTNT)

Was prepared from cadmium acetate and t-nonanethiol following a previously reported procedure^33^.

#### Film Processing

Unless otherwise noted, films were prepared by dissolving metal TAMTs in THF at a concentration of 100 mg/mL, filtering the solution through a 0.45 μm PTFE filter and spin casting at 1500 rpm. Films were then annealed on digital hotplates, in a nitrogen filed glovebox at temperatures in the range of 200–400 °C for 10 minutes to facilitate conversion of the TAMTs to sulfide films.

#### Film Characterization

TGA traces of each sample were collected using a TGA instrument, Q500. To collect each trace, ~20 mg samples were put in aluminium pans and the temperature was raised from 25 to 450 °C at 5 °C/min. XRD patterns were collected using an X-ray diffractometer D/MAX2500V/PC (Rigaku, Japan). Powder samples for TGA and XRD were prepared through annealing of TAMT complexes at 300 °C in a vial under vacuum. Gas chromatography-mass spectrometry (GC-MS) spectra were gathered using Bruker 450-GC & 320-MS. For the measurement, samples were prepared as described in the main text. UV-vis spectrometry was performed using a Varian Carry 5000 spectrophotometer in the range 300 to 1200 nm with 1 nm resolution. AFM images were collected using a Veeco Multimode microscope with 300 kHz silicon tips operating in tapping mode.

SEM images were gathered using NanoSEM 230, FEI. For Raman measurements, sample was prepared by heating an equimolar mixture of Cu and In thiolates to 300 °C under vacuum and collecting the volatile byproducts using a liquid nitrogen trap and dissolving this material in chloroform. Raman spectra were additionally collected for CuInS_2_ film samples prepared as described in the text. Raman spectra were measured using 532 nm excitation wavelength with a WITec alpha300R confocal Raman microscope. Laser beam power was 1 mW and the collected spectra were integrated 10 times.

#### UPS/XPS Measurements

50 nm thick gold films were deposited on pre-cleaned Si substrates with a thin native oxide. Solutions were spin cast onto the gold films using the same procedures used to fabricate devices, as described in the text and device fabrication sections. UPS and XPS experiments were carried out using a Thermo Fisher Scientific ESCALAB 250XI which was maintained at a base pressure of 1.0 × 10^−9^ Torr. UPS measurements were carried out using a He I (hv = 21.2 eV) source while XPS measurements were performed using a monochromated Al-Kα X-ray source. UPS and XPS spectra were collected for films annealed at both 300 and 400 °C, but did not show significant differences.

#### Device fabrication and testing

Glass/ITO substrates were cleaned with detergent, then ultra-sonicated in acetone and isopropanol and subsequently dried in an oven overnight at 100 °C. Metal sulfide films were processed from solutions of each TAMT in THF as described above. In the case of CdS, a solution of CdTNT in chloroform (6 mg/mL) was used. ZnO films were deposited by diluting 200 µL of a solution of diethyl zinc in toluene (Aldrich, 15 wt%) with 400 µL of anhydrous THF, filtering the diluted solution through a 0.45 um PTFE filter and spin coating this solution in air at 3000 rpm, followed by annealing in air at 110 °C for 10 min. Completed devices were pumped down in vacuum (<10^–6^ torr; 1 torr ~133 Pa), and 100 nm thick Al electrodes or 80 nm thick Au electrodes were deposited on top of the active layer by thermal evaporation. J-V measurements were carried out using a Keithley 2635A source measurement unit with the solar cells inside a nitrogen filled glove box using a high quality optical fiber to guide the light from a Xenon arc lamp to the devices. An aperture with an area of 13 mm^2^ defined the active area of the devices. The J-V were measured under AM 1.5G illumination at 100 mWcm^−2^ calibrated with a Si photodiode (PV Measurements, Inc.) which was standardized at the National Renewable Energy Laboratory in Golden, Colorado (US). Reported J-V curves represent the best devices for each condition tested. EQE measurements were conducted in ambient air using an EQE system (Model QEX7) by PV measurements Inc. (Boulder, Colorado). Spectral mismatch factors between the simulator and AM1.5G spectrum were found to be 10% or less.

## Additional Information

**How to cite this article**: Heo, J. *et al.* Clean thermal decomposition of tertiary-alkyl metal thiolates to metal sulfides: environmentally-benign, non-polar inks for solution-processed chalcopyrite solar cells. *Sci. Rep.* **6**, 36608; doi: 10.1038/srep36608 (2016).

**Publisher’s note:** Springer Nature remains neutral with regard to jurisdictional claims in published maps and institutional affiliations.

## Supplementary Material

Supplementary Information

## Figures and Tables

**Figure 1:**
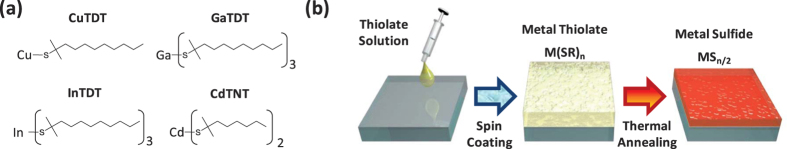
Schematic diagrams including (**a**) TAMT precursor molecules used to prepare sulfide films and (**b**) processing strategy.

**Figure 2:**
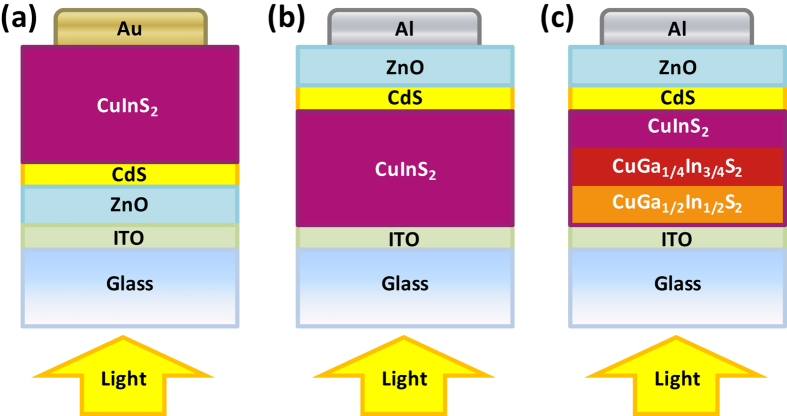
Schematic diagrams of architectures used for (**a**) n-type substrates, (**b**) p-type substrates and (**c**) p-type substrates using an active layer with a graded band-gap.

**Table 1:** Absorption onsets, optical band gaps and band energies of sulfide films.

Material	λ_onset_ (nm)	E_g (opt)_ (eV)	E_VB_ (eV)	φ (eV)	E_CB_ (eV)
Cu_2_S	1020	1.22	4.9	4.9	3.7
In_2_S_3_	550	2.25	6.4	4.9	4.1
Ga_2_S_3_	380	3.26	7.4	4.9	4.1
CuInS_2_	860	1.44	5.4	4.9	4.0
Cu(In,Ga)S_2_ [In:Ga = 3:1]	800	1.55	5.4	4.9	3.9
Cu(In,Ga)S_2_ [In:Ga = 2:2]	720	1.72	5.4	4.9	3.8
