# Supplementary material for: Clean thermal decomposition of tertiary-alkyl metal thiolates to metal sulfides: environmentally-benign, non-polar inks for solution-processed chalcopyrite solar cells
Source: Sci Rep. 2016 Nov 9;6:36608. doi: 10.1038/srep36608 (PMC5101475; doi:10.1038/srep36608)
Supplement: Supplementary Information [file srep36608-s1.pdf]

Supplementary information for: Clean thermal decomposition of tertiary-alkyl metal thiolates to metal sulfides: environmentally-benign, non-polar inks for solution-processed chalcopyrite solar cells

Jungwoo Heo[1], Gi-Hwan Kim[2], Jaeki Jeong[2], Yung Jin Yoon[2], Jung Hwa Seo[3], Bright Walker[2,*] and Jin Young Kim[2,*]

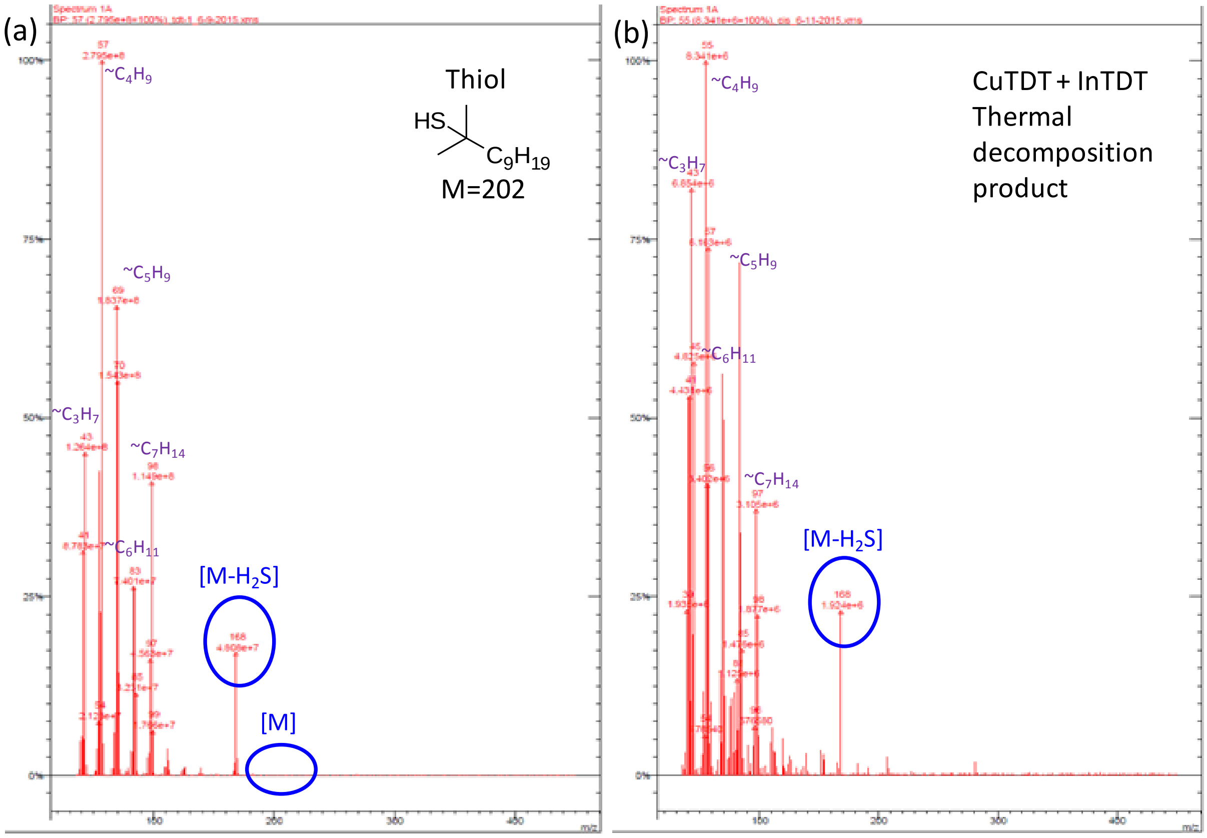

Supplementary Figure S1. Mass spectra of (a) t-dodecanethiol reference and (b) the volatile decomposition products obtained upon heating a 1:1 molar ratio of CuTDT and InTDT to 300 ^oC.

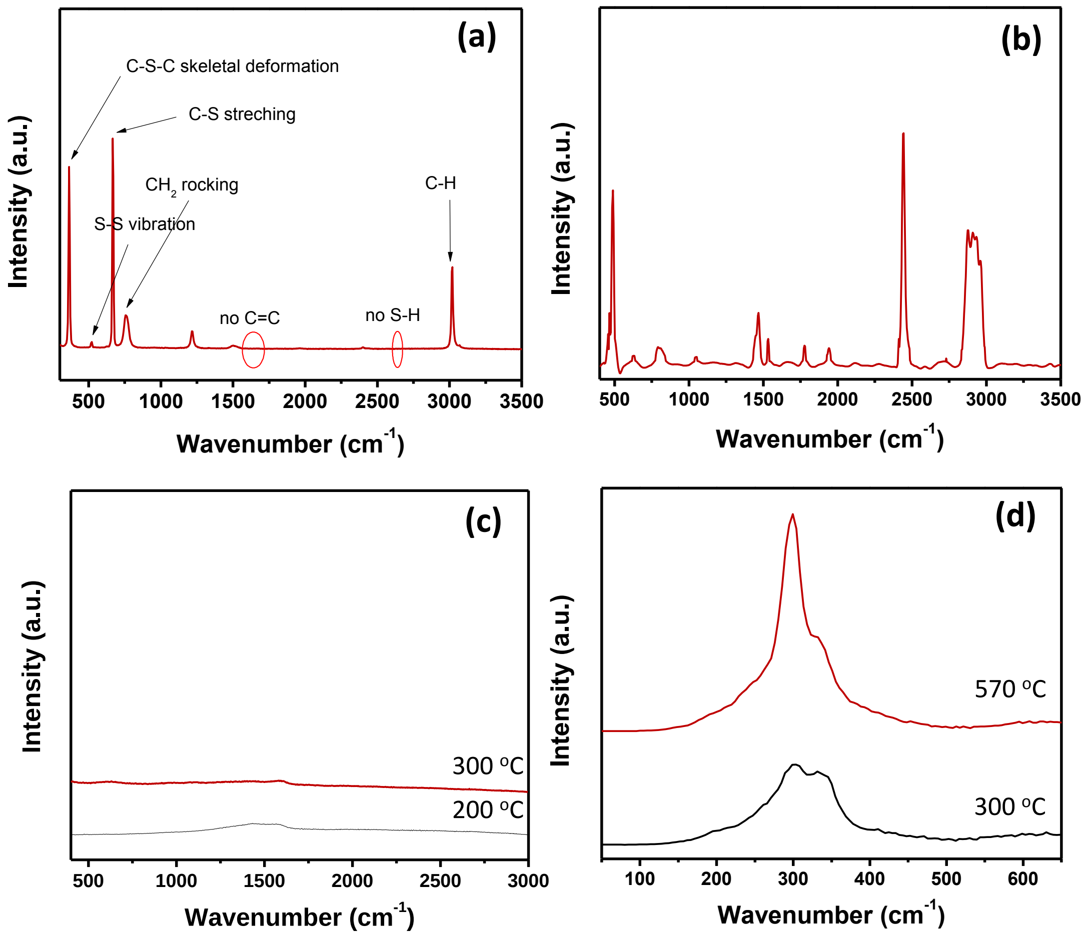

Supplementary Figure S2. Raman spectra taken from (a) $CuInS_2$ volatile decomposition products dissolved in chloroform, (b) TAMT precursor in chloroform, (c) $CuInS_2$ films processed from TAMTs after annealing at 200 and 300 °C, and (d) Comparison of A_1 vibrational mode of films processed at 300 °C, before and after sintering in a vacuum tube furnace at 570 °C.

Supplementary Table S1. Raman vibrational mode assignment for 1-Dodecanethiol.[1]

Mode assignment	Wavenumber (cm^{-1})
$\nu(C\text{-}S)_G$	659
$\nu(C\text{-}S)_T$	735
$\nu(C\text{-}C)_T$	1061-1281
$\nu(C\text{-}C)_G$	1083
$\nu_s(CH_2)$	2848-2857
$\nu_s(CH_3)$	2873
$\nu_a(CH_2)$	2882-2891

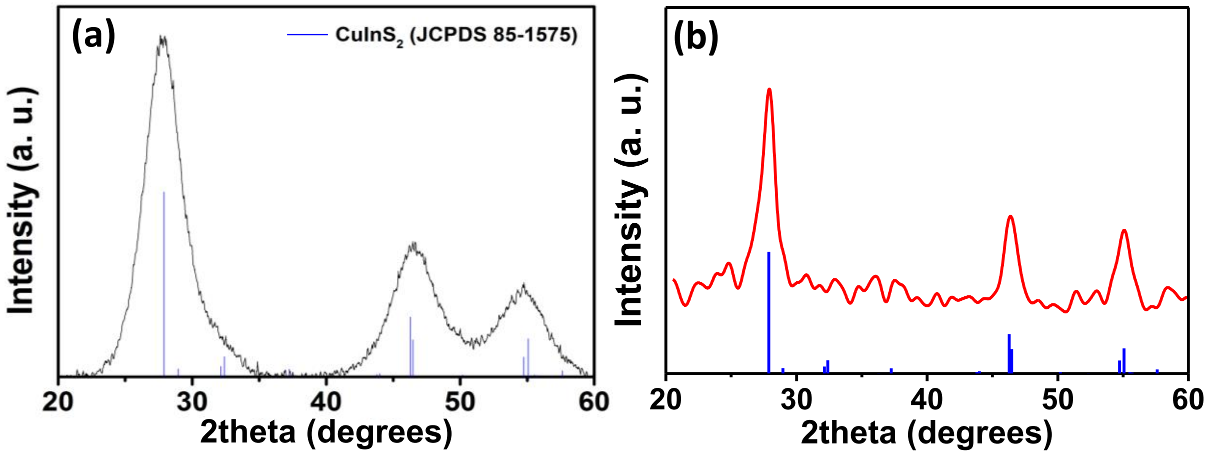

Supplementary Figure S3. X-ray diffraction patterns of $CuInS_2$ materials. (a) Powder annealed at 400 °C. (b) Thin film annealed at 570 °C under a stream of argon.

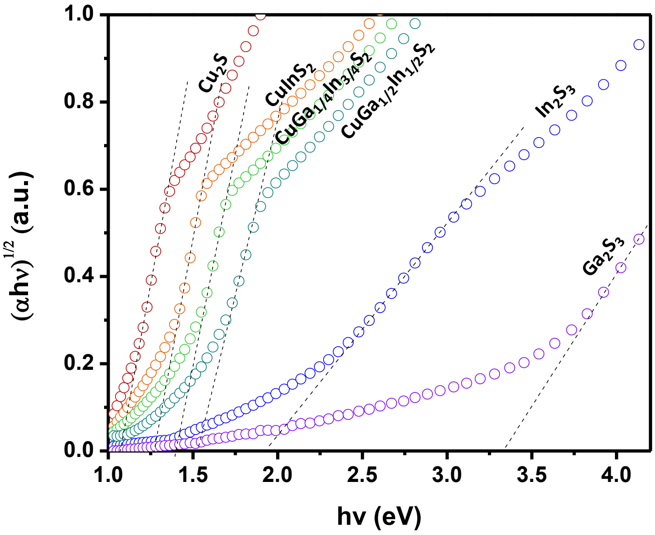

Supplementary Figure S4. Tauc plots ($(\alpha h\nu)^{½}$ vs. hν) for indirect transition of various chalcogenide films annealed at 400 °C.

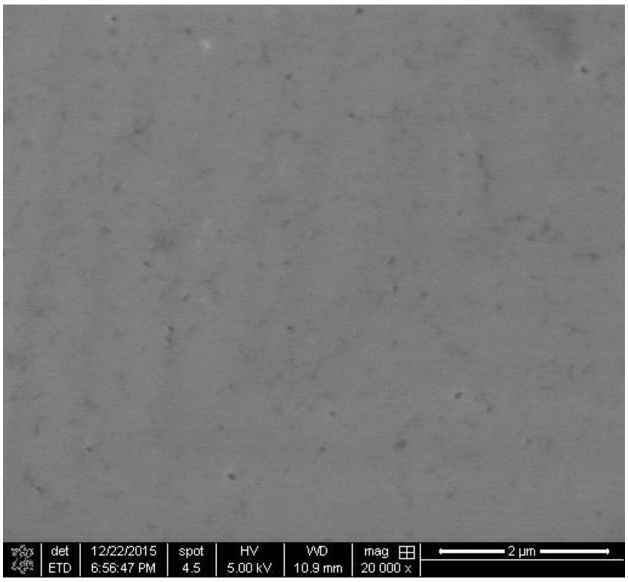

Supplementary Figure S5. SEM image of the surface of a $CuInS_2$ film prepared by annealing an equimolar ratio of CuTDT and InTDT at 300 °C.

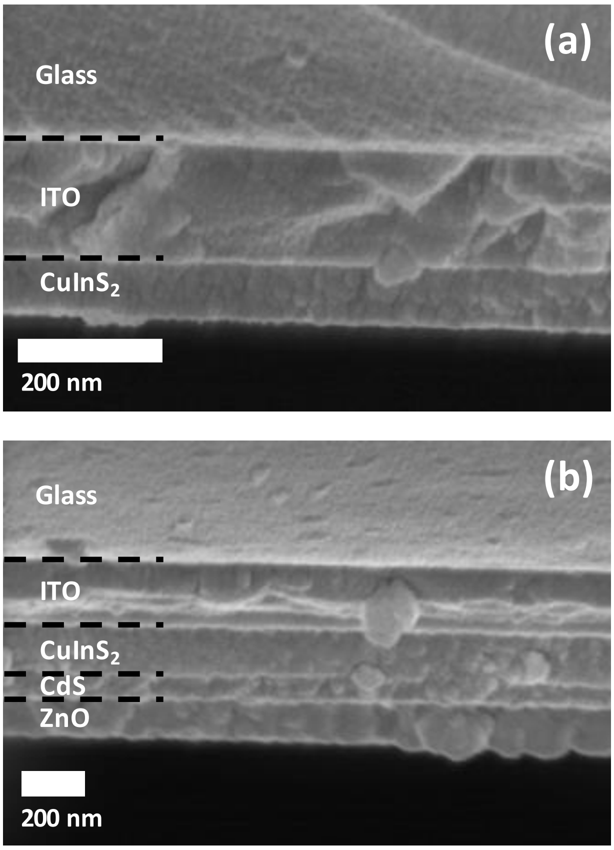

Supplementary Figure S6. SEM images showing (a) a $CuInS_2$ film deposited on a glass/ITO substrate and (b) a device stack consisting of a $CuInS_2$/CdS/ZnO trilayer structure deposited on a glass/ITO substrate. $CuInS_2$ and CdS layers were annealed at 300 °C, while ZnO was annealed at 120 °C.

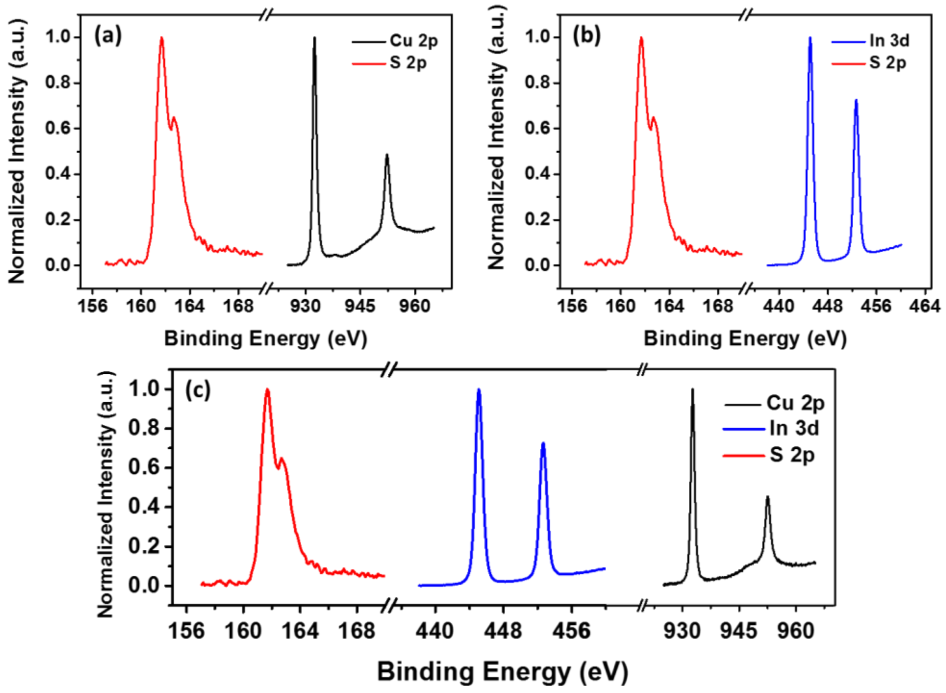

Supplementary Figure S7. XPS spectra collected for (a) Cu_2S, (b) In_2S_3 and (c) $CuInS_2$ films annealed at 300 °C.

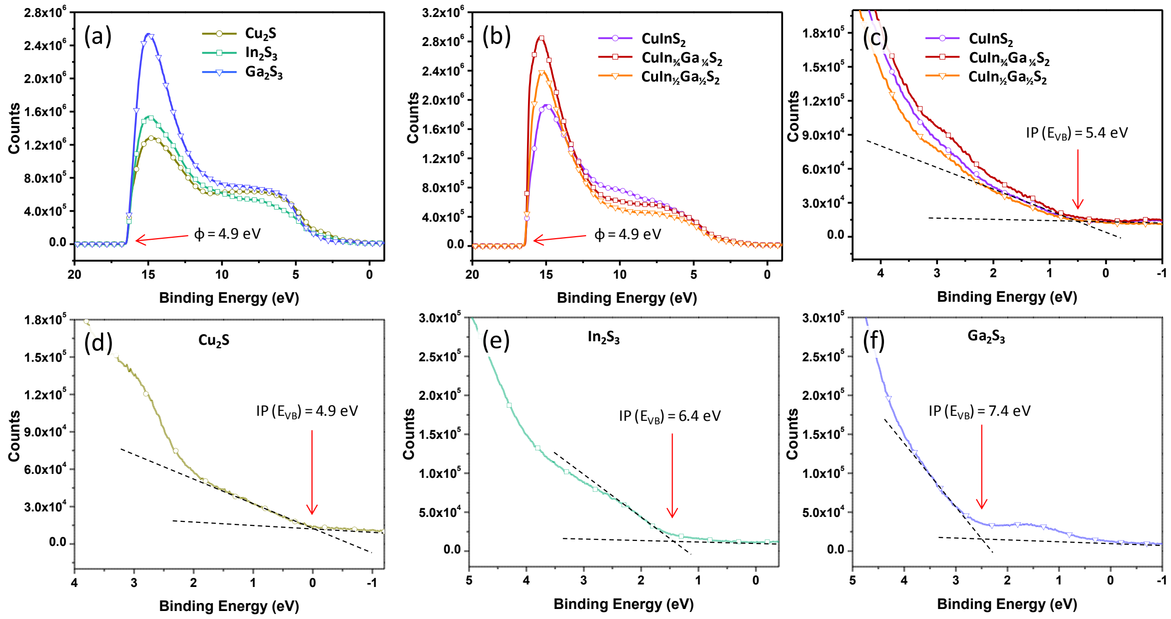

Supplementary Figure S8. UPS spectra collected on Au substrates with a measured work function of 4.9 eV. (a) Overview of Cu_2S, In_2S_3, and Ga_2S_3 spectra. (b) Overview of $CuInS_2$, $CuIn_{3/4}Ga_{1/4}S_2$ and $CuIn_{1/2}Ga_{1/2}S_2$ spectra. All of the materials show the same work function as the gold substrates (4.9 eV), indicating no vacuum level shift. (c) Close-up of Fermi edge region of $CuInS_2$, $CuIn_{3/4}Ga_{1/4}S_2$ and $CuIn_{1/2}Ga_{1/2}S_2$ films. (d-f) Close-up of Fermi edge regions of Cu_2S, In_2S_3 and Ga_2S_3 films, respectively. Ionization potentials (IPs) were calculated relative to the work function of the Au substrates. All films were annealed at 300 °C.

References.

1. Bryant, M.A. & Pemberton, J. E. Surface Raman scattering of self-assembled monolayers formed from 1-alkanethiols at silver [electrodes] *J. Am. Chem. Soc.* **113,** 3629-3637 (1991).
